# Photoplethysmography as a Potential Alternative to Electrocardiography for Recording Heart Rate Intervals Used in Variability Analysis

**Published:** 2012

**Authors:** ŞC Mirescu, SW Harden

**Affiliations:** * “Babeş-Bolyai University”, Faculty of Biology and Geology, Cluj-Napoca, Romania; ** Biomolecular Science Center, Burnett School of Biomedical Sciences, College of Medicine, University of Central Florida, Orlando, Florida, USA

**Keywords:** electrocardiography, photoplethysmography, heart rate variability

## Abstract

**Rationale.** This study investigates the use of photoplethysmography (PPG) as a comfortable alternative to electrocardiography (ECG) for heart rate variability (HRV) analysis. Although HRV is typically analyzed from an ECG trace, PPG poses a likely alternative, as arterial pulsations and peripheral tissue perfusion (as measured by PPG) are coupled with cardiac electrical activity (as measured by ECG). PPG may be more desirable in some clinical circumstances, as a fingertip optical sensor is more comfortable for patients than ECG electrodes, and can reduce waste by not needing to be disposed after each use.

**Objective.** The aim of this study is to assess the efficacy of using PPG as an alternative to ECG for recording cardiac activity for HRV analysis by comparing HRV data obtained using both methods.

**Methods and results.** The study was conducted on 10 healthy human subjects, aged 20 to 25 years (4 males, 6 females). Three lead ECG and fingertip PPG traces were simultaneously recorded with a two-channel analog-to-digital converter and HRV parameters were calculated from each data set. The two sets of HRV data simultaneously obtained from the same subjects were compared. The results showed a high correlation between ECG-derived and PPG-derived HRV data (R^2^>0.95). Time-domain parameters, frequency-domain parameters and Poincaré geometry and analysis showed no significant difference between HRV analyses using PPG as compared to ECG for heart beat detection.

**Discussion.** The comparison between the two methods indicates that PPG is a reliable instrument to precisely assess HRV parameters, showing no statistically significant differences when compared to HRV assessments calculated using ECG.

## Introduction

**Fig. 1:**
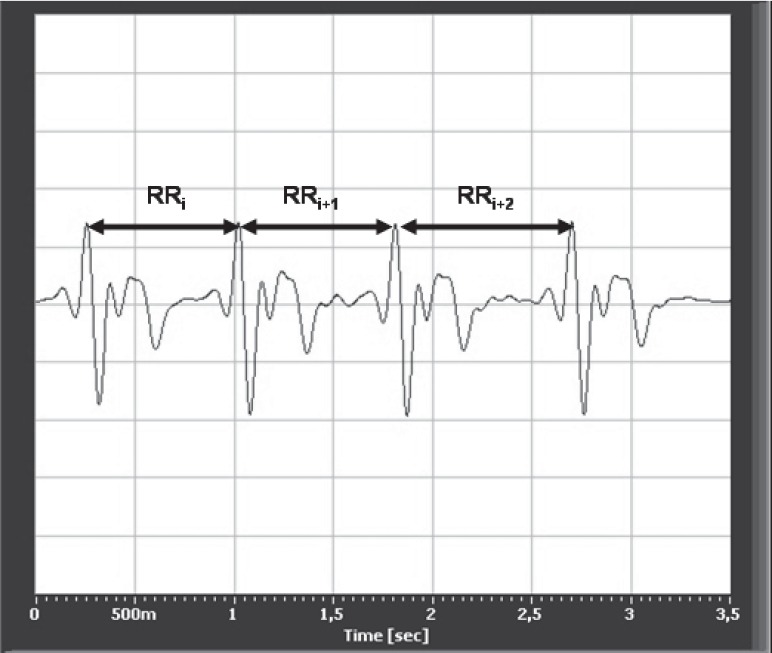
A normal ECG trace

Heart rate variability (HRV) analysis is a common procedure for assessment of autonomic control over the cardiovascular system. Differences in the beat-to-beat pacing intervals (RRIs) of the heart rate are of clinical relevance for both diagnostic and prognostic purposes as they are directly influenced by the sympathetic and parasympathetic branches of the autonomic nervous system [**1**]. Typically, HRV is determined by measuring RRIs from the electrocardiogram (ECG) trace by measuring electric potential across the chest (**Fig. 1**) from which RRIs are calculated (reported in milliseconds) and plotted in sequence (the time of one RRI with respect to the previous RRI). Thus, HRV is generally measured by electrical activity and not by mechanical activity [**2**].

**Fig. 2:**
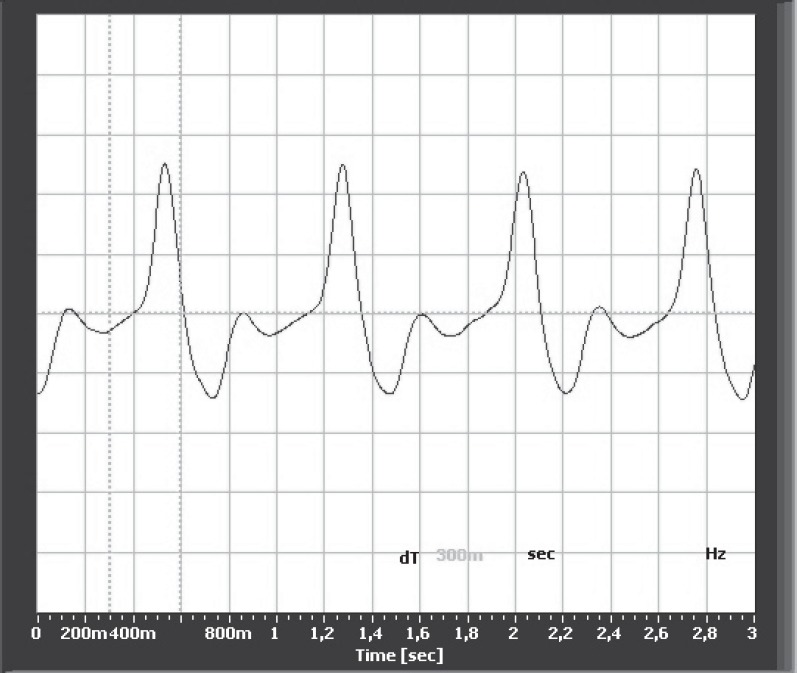
A normal PPG trace

Photoplethysmography (PPG) is an optical technique that typically operates using infrared light, allowing transcutaneous registration of venous and/or arterial blood volume changes in the skin vessels. The complex interaction between the heart and connective vasculature are the components of the mechanism that generate the PPG signal [**3**]. PPG is a non-invasive method of detecting cardiovascular pulse waves that propagate through the vasculature of the body (**Fig. 2**). Compared to the other types of plethysmography, PPG is easy to set up, simple to use, low in cost (no disposable components are necessary) and requires no patient preparation [**4**]. The most well known application of PPG is pulse oximetry monitoring, which uses the difference between the absorption of red and infrared light by the blood to calculate the oxygen saturation of arterial hemoglobin [**5**].

The pulse travel time (the difference between the R wave in an ECG and the peak of the corresponding beat as measured by PPG) shows very minor (millisecond level) beat-to-beat fluctuations, so that the heartbeat intervals derived from ECG and PPG are very similar, but they are not exactly the same [**1**].

This exhaustive study records, calculates, and describes a wide range of HRV parameters derived from a PPG signal, and compares these data to the same parameters obtained from a simultaneously recorded ECG. The aim of the study is to evaluate the differences between ECG and PPG derived HRV data, in order to certify the use of PPG for HRV analysis.

## Methods

The study was conducted on 10 healthy human subjects, aged 22-25 years (4 males, 6 females). The Babes-Bolyai University Official Board of Ethics approved the study. Each participating patient signed a written informed consent agreement and all procedures followed hospital and academic guidelines for human subjects handling.

**Fig. 3:**
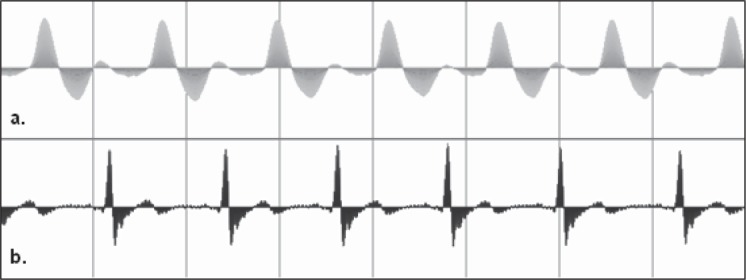
Simultaneous two-channel recording of PPG trace (a) and ECG trace (b)

Signals were recorded with a two-channel analog-to-digital converter (one channel for ECG and one for PPG – **Fig. 3**). ECG was recorded from each subject using two chest electrodes and a right leg drive [**6**]. Adhesive hypoallergenic AgCl electrodes were used for ECG recording. The ECG signal was amplified with an AD620 operational amplifier. To improve the quality of signals by reducing the effects of high frequency electronic interference, a 30 Hz low-pass filter was consecutively applied.

PPG was measured using two optical infrared diodes (wavelength of 830 nm). The signal captured by the receiver photodiode was amplified and recorded.

Subjects were placed comfortably in sitting positions, with the electrodes in place and the finger clip sensor attached to the index of the left hand. To reduce the impact of trace artifacts, subjects were asked not to move or speak during the duration of all experiments. Each participant was subjected to a five minutes long recording.

The two channels were separately processed. Individual beats were identified from the trace data using an automated heartbeat detection script and HRV parameters were calculated with *Kubios Heart Rate Variability Analysis Software*, and a beat-to-beat tachogram was constructed.

The following HRV measures were computed from the ECG and PPG signals, respectively (**Table 1**):

1. Time-domain parameters: average of the RRIs, standard deviation of the RRIs, average of heart rate, standard deviation of heart rate, the root mean square of the squared differences (RMSSD) between successive RRIs, the number of interval differences of successive RRIs greater than 50 ms (NN50) and the percent proportion of successive RRIs greater than 50 ms (pNN50);
2. Frequency-domain parameters: normalized low frequency (LF) and high frequency (HF) band powers;
3. Poincaré plot parameters: standard deviations of short axis (SD1) and standard deviation of long axis (SD2).

The results were also displayed in graphical format, permitting a visual and geometrical comparison of the two processed signals.

Statistical processing was accomplished by using *Microsoft Excel* and *GraphPad*.

## Results

**Fig. 4:**
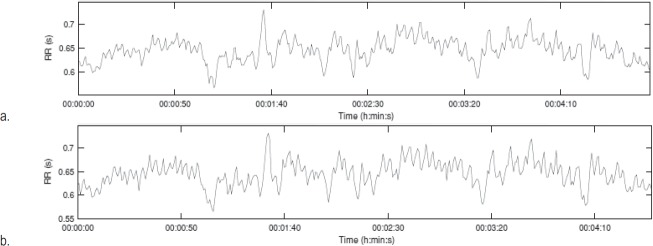
Tachogram obtained from ECG (a) and PPG (b)

**Fig. 5:**
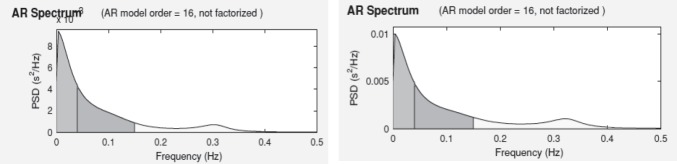
AR spectrum obtained from ECG (left) and PPG (right)

**Fig. 6:**
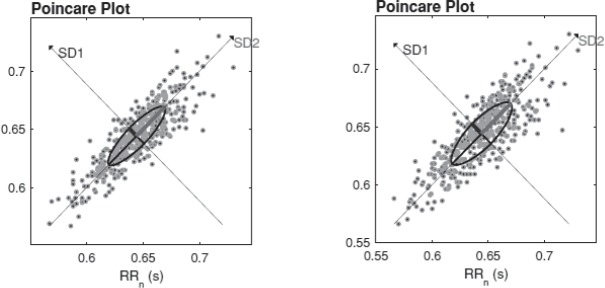
Poincaré plot obtained from ECG (left) and PPG (right)

**Fig. 4** **-** **6** express the HRV parameters derived from ECG and PPG, respectively, for a representative subject (female, 23 years old). A good geometrical match is observed between the tachograms, AR spectrum diagrams and the Poincaré plots obtained from ECG and PPG, for the same analyzed subject.

**Table 1.** Comparison between HRV parameters derived from ECG and PPG

	**Obtained from ECG**			**Obtained from PPG**			**R^2^ value**
***Time-series parameters***							
Average of RR intervals (ms)	718.8	±	91.5	718.9	±	91.9	0.99
Standard deviation of RR intervals (ms)	43	±	17	44	±	16	0.99
Average heart rate (bpm)	84	±	9	84	±	9	0.99
Standard deviation of heart rate (bpm)	5	±	1	5	±	1	0.99
RMSSD (ms)	28	±	15	33	±	17	0.99
NN50 (count)	58	±	81	59	±	72	0.95
pNN50 (%)	10	±	15	13	±	16	0.98
***Frequency-domain parameters***							
Normalized LF power (n.u.)	63	±	11	58	±	10	0.97
Normalized HF power (n.u.)	36	±	11	41	±	10	0.98
***Poincaré plot parameters***							
SD1 (ms)	20	±	11	23	±	12	0.98
SD2 (ms)	57	±	22	58	±	11	0.99

There were no statistically significant differences concerning gender in the present study.

## Discussion

Results demonstrate that PPG may serve as an alternative to ECG in evaluating HRV geometry and parameters. All studied categories of HRV analysis parameters expressed high similarity between the two methods. Therefore, we conclude that PPG is a reliable instrument for recording heart beat intervals used for HRV analysis. As a potential alternative to ECG analysis, PPG measurement to assess HRV is comfortable for the patient, convenient and inexpensive for medical staff, it does not require highly qualified personnel for device and patient preparation, and it does not require disposable materials to operate. All these characteristics suggest PPG may be a suitable alternative to ECG for the recording and calculation of HRV parameters.

## Acknowledgements

Many thanks to *SC Tehnomed Aparatură Electronică şi Tehnică Medicală*, for providing the disposable electrodes needed for ECG recordings.
